# Major systemic infection following breast cancer surgery and oncological outcomes

**DOI:** 10.1093/bjs/znaf233

**Published:** 2025-12-02

**Authors:** Linda Adwall, Irma Fredriksson, Hella Hultin, Peter Stålberg, Maria Mani, Olov Norlén, Helena Sackey

**Affiliations:** Department of Surgery, Division of Breast Surgery, Södersjukhuset, Stockholm, Sweden; Department of Surgical Sciences, Uppsala University, Uppsala, Sweden; Department of Molecular Medicine and Surgery, Karolinska Institutet, Stockholm, Sweden; Department of Breast, Endocrine Tumors and Sarcoma, Karolinska Comprehensive Cancer Center, Karolinska University Hospital, Stockholm, Sweden; Department of Molecular Medicine and Surgery, Karolinska Institutet, Stockholm, Sweden; Department of Surgical Sciences, Uppsala University, Uppsala University Hospital, Uppsala, Sweden; Department of Surgical Sciences, Section of Plastic Surgery, Uppsala University, Uppsala University Hospital, Uppsala, Sweden; Department of Surgical Sciences, Uppsala University, Uppsala University Hospital, Uppsala, Sweden; Department of Molecular Medicine and Surgery, Karolinska Institutet, Stockholm, Sweden; Department of Breast, Endocrine Tumors and Sarcoma, Karolinska Comprehensive Cancer Center, Karolinska University Hospital, Stockholm, Sweden

## Abstract

**Background:**

Postoperative infections are well-known complications following cancer surgery and are associated with worse oncological outcomes in several cancer types. The influence of major systemic postoperative infections on the risk of breast cancer recurrence remains unexplored. The primary aim of this study was to assess the risk of distant recurrence following major systemic infection. Secondary aims were to assess this risk after other major events such as stroke, myocardial infarction and pulmonary embolism.

**Methods:**

This nationwide cohort study included patients who underwent breast cancer surgery in Sweden between 2008 and 2019. The study cohort was identified using BCBaSe 3.0, a database linking the Swedish National Breast Cancer Quality Register with other national population-based healthcare registers. The primary exposure was major systemic infection within 90 days of surgery, with a secondary analysis of other major events. The primary outcome was distant recurrence, whereas secondary outcomes included locoregional recurrence, overall survival, and breast cancer-specific survival.

**Results:**

Among 82 102 patients included, 1.8% (*n* = 1461) experienced a major systemic infection, and 0.6% (*n* = 516) other major events within 90 days of surgery. In adjusted analyses, major systemic infection was associated with increased risk of distant recurrence (HR 1.23, 95% c.i. 1.07–1.41), overall death (HR 1.47, 1.32–1.64), breast cancer-specific death (HR 1.27, 1.06–1.51), but not with locoregional recurrence.

**Conclusions:**

At a median follow-up of 4.8 years, major systemic postoperative infections were associated with an increased risk of distant recurrence, overall death, and breast cancer-specific death, highlighting the importance of timely and effective treatment of postoperative infections.

## Teaser

This nationwide cohort study, including 82 102 patients, showed an association between postoperative major systemic infections after breast cancer surgery and distant recurrence, overall death, and breast cancer-specific death. This highlights the importance of timely and effective treatment of postoperative infections.

## Introduction

Postoperative infections have been linked to worse oncological outcomes across several cancer types^1–11^, but evidence regarding breast cancer remains conflicting^1–6,12–17^. In a recently published population-based study, no significant association between surgical site infection (SSI) following breast cancer surgery and risk of recurrence or breast cancer-specific death was found^18^.

Surgery can suppress cell-mediated immunity through local and systemic physiological responses. Tissue damage activates the sympathetic nervous system and endocrine stress responses. Additionally, tissue damage and associated inflammation trigger the local release of prostaglandins and catecholamines^19–22^. The degree and duration of immunosuppression correlates with the extent of surgery^19^. It may take weeks to months for the immune system to fully recover and during this period, patients are more susceptible to potentially life-threatening infections such as pneumonia and sepsis^19^. In light of these considerations, it is conceivable that major postoperative complications, such as systemic infections, pulmonary embolism, stroke, or myocardial infarction, could stimulate subclinical micrometastases and promote cancer recurrence due to a secondary inflammatory response^23,24^.

The primary aim of this study was to evaluate the risk of distant breast cancer recurrence following major systemic infection. Secondary aims were to assess the risk of distant breast cancer recurrence associated with other major events, as well as to evaluate the risk of locoregional recurrence (LRR), overall death, and breast cancer-specific death for all exposures.

## Methods

### Data source

The study was based on data from the Breast Cancer Database Sweden 3.0 (BCBaSe 3.0), described in detail^18^. Briefly, BCBaSe 3.0 is a mega-linkage cohort including individuals diagnosed with breast cancer in Sweden from 2008 to 2019. It was created to facilitate population-based epidemiological breast cancer research through the integration of individual-level data from the Swedish National Breast Cancer Quality Register (NKBC) with national demographic and population-based healthcare registers maintained by the Swedish National Board of Health and Welfare, Statistics Sweden, and the Swedish Social Insurance Agency (*Fig. S1*).

NKBC includes detailed clinical data on patient and tumour characteristics, treatment, and follow-up. Its completeness is high (>99%), verified through cross-linkage to the National Cancer Register, to which reporting is mandatory by law^25^. Most variables have <5% missing values and high agreement with original medical chart data^25^. The National Patient Register contains information on both in- and outpatient patient care, including up to eight discharge diagnoses classified according to ICD. It also records information on surgical procedures, as well as admission and discharge dates. The register is estimated to cover approximately 99% of all hospitalizations^26^. Further information about the registers linked in BCBaSe 3.0, their validity and coverage are presented in *Supplementary methods*.

In the current study, follow-up for distant recurrence and survival analysis began 90 days after primary surgery, whereas follow-up for LRR started one year after primary surgery. Follow-up was continued until death or the end of the follow-up period on 31 December 2019.

This article was written in accordance with the STROBE guidelines^27^.

### Patients

This large population-based cohort study includes several substudies using the same study base, where the effect of minor SSI, major SSI, and major systemic infection on breast cancer outcomes is analysed. The study cohort was identified using BCBaSe 3.0 and includes all patients who underwent surgery for primary invasive or ductal carcinoma in situ (DCIS) in Sweden between 1 January 2008 and 30 September 2019. Patients diagnosed with invasive breast cancer or DCIS before January 2008, those with distant metastasis at the time of or within 3 months of primary surgery, and those with distant metastases from other malignancies, were excluded. Patients with synchronous bilateral breast cancer were included once, with the most advanced cancer recorded as the index tumour based on the following criteria: highest tumour (T) stage, highest nodal (N) stage, subtype with more aggressive biology, highest histological grade, and highest Ki67-level.

### Exposures, outcome, and predictors

#### Exposures

For this study, the primary exposure was major systemic infection within 90 days of surgery, defined as a condition requiring inpatient care due to a systemic infection. The secondary exposure was other major events within 90 days of surgery. Major systemic infection was categorized as early (within 30 days of surgery) or within 90 days after surgery. Other major events considered in this study were stroke (ICD-10 codes I61, I63, I64), pulmonary embolism (I26), and/or myocardial infarction (I21, I22).

In the entire study cohort, systemic infection (both minor/major) was defined using the following ICD-10 codes: N39.0, N30.9 (urinary tract infection), J09–J18 (pneumonia), J03 (tonsillitis), K57 (diverticulitis), L08, L03, L01, A46 (skin infection), A40, A41 (sepsis), J01 (sinusitis), H66 (otitis), I33, I38, I39 (endocarditis), I40, I41 (myocarditis), G04, G05 (encephalitis), G00, G01, G02, G03 (meningitis), M86 (osteomyelitis), N10 (acute pyelonephritis), J00, J02, J04–J06 (upper respiratory infection), J20–J22 (lower respiratory infection), B02 (herpes zoster), A00–A09 (gastroenteritis), B15, B16, B17 (acute hepatitis), L04 (acute lymphadenitis), A15–A19 (tuberculosis), A48, A49 (other bacterial infections), A80–A89 (virus infection of the central nervous system), B33, B34, B99 (unspecified virus infection), A69.2 (Lyme disease). Alternatively, it was defined as the dispensation of antibiotics classified as J01 within 90 days of surgery, excluding flucloxacillin (J01CF05) and clindamycin (J01FF01) (used for SSI). In addition, ciprofloxacin (J01MA02) was excluded for patients who received adjuvant chemotherapy between 2008 and 2011, as ciprofloxacin was used as antibiotic prophylaxis within the Panther study^28^, recruiting patients during these years. In this study focusing on major systemic infections, patients treated exclusively with antibiotics commonly used for minor surgical site infections (for example flucloxacillin or clindamycin) were considered unexposed. Additionally, patients with systemic infections that did not require inpatient care were also considered unexposed.

#### Outcome

The primary outcome was distant recurrence of breast cancer, defined as ICD codes C780–C788, C790–C791, C793–C799, C771, C772, C778 (definition of ICD codes available in *Supplementary methods*), and/or breast cancer death occurring more than 3 months after primary surgery. Only patients with invasive breast cancer were included in this analysis.

LRR, overall survival (OS), and breast cancer-specific survival (BCSS) were secondary outcomes. LRR was defined as recurrence in the ipsilateral breast or regional lymph nodes (ICD codes C50, D05 (excluding D05.0 lobular carcinoma in situ), or C792, C770, C773, C779 (definition of ICD codes available in *Supplementary methods*), and/or breast radiotherapy (RT) to the ipsilateral breast more than 1 year after primary surgery and/or ICD intervention codes for ipsilateral breast cancer surgery in the breast and/or axilla (HAB00, HAB40, HAB99, HAC10, HAC15, HAC20, HAC22, HAC99, PJA10, VXA20, PJA42, VXK21, HAF00, HAF99) performed more than 1 year after primary surgery, excluding secondary reconstructive procedures (without diagnosis code C50 or D05).

#### Predictors

Predictors included age at surgery, country of birth, highest level of education (9 years or less (primary), 10–13 years (secondary), or >13 years (tertiary)), family income (low (Q1: 0–25%), middle (Q2–Q3: >25–75%), or high (Q4: 75–100%)), menstrual status, hypertension, obesity, diabetes, autoimmune disease, immunodeficiency, Charlson Comorbidity Index (CCI), breast cancer detection mode, breast cancer laterality, year of breast cancer surgery, region of residence at surgery, type of primary treatment (surgery or neoadjuvant therapy (NAT)), type of final breast surgery, type of final axillary surgery, number of surgeries, RT, time to RT, chemotherapy, endocrine therapy, anti-human epidermal growth factor receptor 2 (anti-HER2) therapy, invasivity (invasive/*in situ*), T stage, N stage, histological tumour type, Nottingham histological grade, oestrogen receptor, progesterone receptor, HER2, Ki67, and breast cancer subtype by proxy. Detailed information and definitions of the predictors have been published previously^18^.

### Statistical analysis

Descriptive statistics were reported as numbers with percentages and means (s.d.). Distant recurrence-free survival (DRFS), LRR, OS, and BCSS were estimated using the Kaplan–Meier method, and univariable analyses of the effect of exposure were analysed with the log-rank test. A directed acyclic graph was constructed to identify confounders to adjust for in the multivariable analysis. Associations between exposure, predictors, and outcomes were analysed using multivariable Cox regression. The model was adjusted stepwise. First, for patient characteristics (age, year of surgery, and region of residence), second also for disease characteristics (T stage, subtype, N stage, and histological tumour type) and third adding co-morbidity (CCI). In the fourth model, socioeconomic factors (country of birth, highest level of education, and family income) was added, in the fifth surgical treatment (type of primary treatment, final breast/axillary surgery, and number of surgeries), and in the full model also oncological treatment (RT, chemotherapy, endocrine therapy, and anti-HER2 therapy).

A sensitivity analysis was performed, excluding all patients with any malignancy prior to breast cancer diagnosis. The results are presented as hazard ratios with 95% confidence intervals.

All analyses were conducted using R version 4.3.1 (R Foundation for Statistical Computing, Vienna, Austria), utilizing specific study files accessible via ‘remote server access’ on the research Q-portal managed by the Regional Cancer Centre North. All tests were two-sided, and a *P* less than 0.05 was considered statistically significant.

### Ethical considerations

The context of BCBaSe 3.0, which was approved by the Regional Ethical Committee (DNR 2019–02610, 2020-00886, 2020-06302) with the addition of the present study (DNR 2022-01020-02).

## Results

**Fig. 1:**
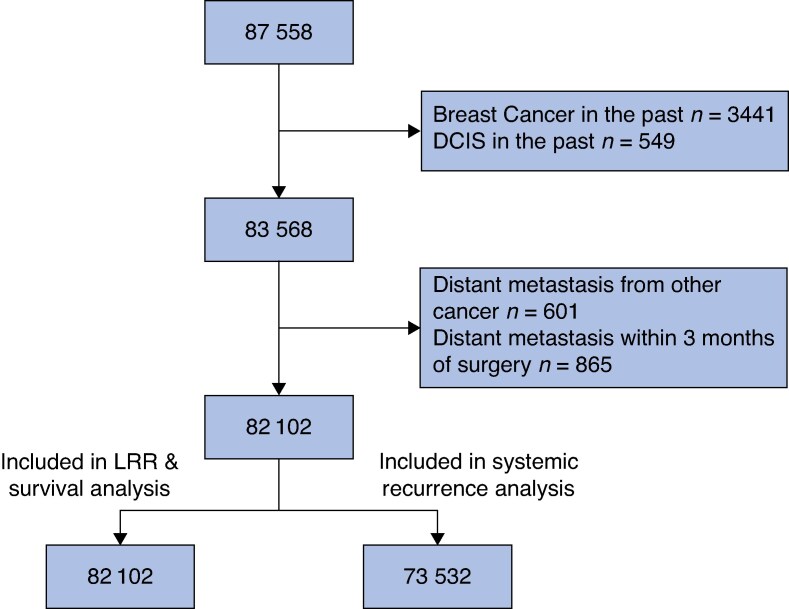
Flow chart of the study cohort

A total of 87 558 patients met the inclusion criteria. Patients with a history of previous invasive breast cancer or DCIS, distant metastases from other cancers, or distant metastasis within 3 months of breast cancer surgery were excluded, resulting in a final study cohort of 82 102 patients (*Fig. 1* ), of whom 513 (0.6%) were men. The patient characteristics are detailed in *Table 1*, the treatment characteristics in *Table S1*, and the disease characteristics in *Table 2*. The mean age was 63 years (s.d. 13), ranging from 19 to 104 years. The cohort comprised 73 313 patients with invasive breast cancer and 8 570 with DCIS. The median follow-up was 4.8 years (range 0–11.8) for distant recurrence, 4.5 years (0–11.0) for LRR and 5.0 years (0–11.8) for survival analysis.

**Table 1:** Patient characteristics and occurrence of major systemic infection within 90 days in a population-based cohort of 82 102 individuals diagnosed with breast cancer between 2008 and 2019

	No infection *n* = 80 641 (%)	Infection *n* = 1 461 (%)	Overall *n* = 82 102 (%)
**Age group (years)**			
<40	3029 (3.8)	68 (4.7)	3097 (3.8)
40–49	12 277 (15.2)	174 (11.9)	12 451 (15.2)
50–64	28 356 (35.2)	461 (31.6)	28 817 (35.1)
65–74	24 810 (30.8)	415 (28.4)	25 225 (30.7)
≥75	12 169 (15.1)	343 (23.5)	12 512 (15.2)
**Country of birth**			
Sweden	68 599 (85.1)	1228 (84.1)	69 827 (85.1)
Europe (excluding Sweden)	6483 (8.0)	140 (9.6)	6623 (8.1)
Outside Europe	3251 (4.0)	53 (3.6)	3304 (4.0)
Missing	2308 (2.9)	40 (2.7)	2348 (2.9)
**Highest level of education**			
≤9 years	17 360 (21.5)	399 (27.3)	17 759 (21.6)
10–13 years	33 553 (41.6)	599 (41.0)	34 152 (41.6)
>13 years	28 842 (35.8)	449 (30.7)	29 291 (35.7)
Missing	886 (1.1)	14 (1.0)	900 (1.1)
**Family income**			
Low	19 927 (24.7)	471 (32.2)	20 398 (24.8)
Middle	40 233 (49.9)	694 (47.5)	40 927 (49.9)
High	20 230 (25.1)	296 (20.3)	20 526 (25.0)
Missing	251 (0.3)	0 (0.0)	251 (0.3)
**Menstrual status**			
Premenopausal	16 819 (20.9)	247 (16.9)	17 066 (20.8)
Postmenopausal	57 548 (71.4)	1103 (75.5)	58 651 (71.4)
Male	493 (0.6)	20 (1.4)	513 (0.6)
Missing	5781 (7.2)	91 (6.2)	5872 (7.2)
**Hypertension**			
No	63 920 (79.3)	1007 (68.9)	64 927 (79.1)
Yes	16 721 (20.7)	454 (31.1)	17 175 (20.9)
**Obesity**			
No	76 630 (95.0)	1337 (91.5)	77 967 (95.0)
Yes	4011 (5.0)	124 (8.5)	4135 (5.0)
**Diabetes**			
No	74 718 (92.7)	1295 (88.6)	76 013 (92.6)
Yes	5923 (7.3)	166(11.4)	6089 (7.4)
**Autoimmune disease**			
No	74 573 (92.5)	1262 (86.4)	75 835 (92.4)
Yes	6068 (7.5)	199 (13.6)	6267 (7.6)
**Immunodeficiency**			
No	80 470 (99.8)	1459 (99.9)	81 929 (99.8)
Yes	171 (0.2)	2 (0.1)	173 (0.2)
**CCI**			
0	43 951 (54.5)	716 (49.0)	44 667 (54.4)
1	7541 (9.4)	191 (13.1)	7732 (9.4)
2	11 252 (14.0)	232 (15.9)	11 484 (14.0)
3–5	3999 (5.0)	150 (10.3)	4149 (5.1)
6–7	321 (0.4)	16 (1.1)	337 (0.4)
≥8	401 (0.5)	25 (1.7)	426 (0.5)
Missing	13 176 (16.3)	131 (9.0)	13 307 (16.2)

Values are numbers (percent). CCI Charlson comorbidity index. Percentages may not add up to 100% owing to rounding.

**Table 2:** Disease characteristics and occurrence of major systemic infection within 90 days in a population-based cohort of 82 102 individuals diagnosed with breast cancer between 2008 and 2019

	No infection *n* = 80 641 (%)	Infection *n* = 1 461 (%)	Overall *n* = 82 102 (%)
**Invasivity**			
Invasive	71 892 (89.2)	1421 (97.3)	73 313 (89.3)
*In situ*	8537 (10.6)	33 (2.3)	8570 (10.4)
Missing	212 (0.3)	7 (0.5)	219 (0.3)
**Tumour stage**			
T0	483 (0.6)	12 (0.8)	495 (0.6)
Tis	8537 (10.6)	33 (2.3)	8570 (10.4)
T1	44 704 (55.4)	684 (46.8)	45 388 (55.3)
T2	21 798 (27.0)	605 (41.4)	22 403 (27.3)
T3	3502 (4.3)	89 (6.1)	3591 (4.4)
T4	610 (0.8)	14 (1.0)	624 (0.8)
Unknown	1004 (1.3)	24 (1.6)	1028 (1.3)
**Histological tumour type**			
NST	55 037 (68.3)	1134 (77.6)	56 171 (68.4)
NST + lobular	1511 (1.9)	31 (2.1)	1542 (1.9)
Lobular	9222 (11.4)	156 (10.7)	9378 (11.4)
Other invasive	5091 (6.3)	85 (5.8)	5176 (6.3)
*In situ*	8537 (10.6)	33 (2.3)	8570 (10.4)
Missing	1254 (1.5)	22 (1.5)	1265 (1.5)
**Nottingham histological grade**			
1	14 053 (17.4)	129 (8.8)	14 182 (17.3)
2	34 239 (42.5)	574 (39.3)	34 813 (42.4)
3	18 865 (23.4)	614 (42.0)	19 479 (23.7)
*In situ*	8537 (10.6)	33 (2.3)	8570 (10.4)
Missing	4947 (6.1)	111 (7.6)	5058 (6.2)
ER*			
Positive	61 175 (84.8)	1090 (76.3)	62 265 (84.7)
Negative	9681 (13.4)	322 (22.5)	10 003 (13.6)
Missing	1248 (1.7)	16 (1.1)	1264 (1.7)
PR*			
Positive	51 649 (71,6)	872 (61.1)	52 521 (71.4)
Negative	19 124 (26.5)	539 (37.7)	19 663 (26.7)
Missing	1331 (1.8)	17 (1.2)	1348 (1.8)
HER2*			
Positive	9235 (12.8)	298 (20.9)	9533 (13.0)
Negative	59 225 (82.1)	1067 (74.7)	60 292 (82.0)
Missing	3644 (5.1)	63 (4.4)	3707 (5.0)
Ki67*			
Low	19 240 (26.7)	238 (16.7)	19 478 (26.5)
Intermediate	6703 (9.3)	90 (6.3)	6793 (9.2)
High	21 150 (29.3)	617 (43.2)	21 767 (29.6)
Missing	25 011 (34.7)	483 (33.8)	25 494 (34.7)
**Subtype**			
Luminal A	27 235 (33.8)	296 (20.3)	27 531 (33.5)
Luminal B	14 799 (18.4)	380 (26.0)	15 179 (18.5)
HER2+HR+	6306 (7.8)	210 (14.4)	6516 (7.9)
HER2+HR−	2870 (3.6)	86 (5.9)	2956 (3.6)
TNBC	6085 (7.6)	207 (14.2)	6292 (7.7)
*In situ*	8537 (10.6)	33 (2.3)	8570 (10.4)
Missing	14 809 (18.4)	249 (17.0)	15 058 (18.3)
**Subtype (other definition)†**			
Luminal A (NHG 1)	12 997 (16.1)	109 (7.5)	13 106 (16.0)
Luminal NHG 2	29 078 (36.1)	447 (30.6)	29 525 (36.0)
Luminal B (NHG 3)	8953 (11.1)	256 (17.5)	9209 (11.2)
HER2+HR+	6306 (7.8)	210 (14.4)	6516 (7.9)
HER2+HR−	2870 (3.6)	86 (5.9)	2956 (3.6)
TNBC	6085 (7.6)	207 (14.2)	6292 (7.7)
*In situ*	8537 (10.6)	33 (2.3)	8570 (10.4)
Missing	5815 (7.2)	113 (7.7)	5928 (7.2)
**Nodal stage**			
N0	56 541 (70.1)	770 (52.7)	57 311 (69.8)
N1	17 836 (22.1)	466 (31.9)	18 302 (22.3)
N2	4061 (5.0)	158 (10.8)	4219 (5.1)
N3	1760 (2.2)	61 (4.2)	1821 (2.2)
Missing	443 (0.6)	6 (0.4)	449 (0.6)

Values are numbers (percent). *DCIS with data not shown (*n* = 8570). †Due to a large proportion of missing data on Ki67, this subtype definition was used in the multivariable regression analysis. Percentages may not add up to 100% owing to rounding. Tis: carcinoma *in situ*; NST: no special type (former ductal); ER: oestrogen receptor; PR: progesteron receptor; HER2: human epidermal growth factor receptor 2; HR: hormone receptor; TNBC: triple-negative breast cancer; NHG: Nottingham histological grade.

Overall, 1461 patients (1.8%) had a major systemic infection within 90 days of surgery, whereas 348 patients (0.4%) within 30 days. There were 516 patients (0.6%) who experienced a major event (178 patients (0.2%) had a stroke, 262 patients (0.3%) a pulmonary embolism, and 81 patients (0.1%) a myocardial infarction) within 90 days.

For patients not treated with adjuvant chemotherapy, the mean time to RT was 79.7 days (s.d. 42.4), for those who experienced a major systemic infection within 90 days of surgery, compared to 71.9 days (s.d. 31.0) (*P* < 0.001) for those without a major systemic infection.

A total of 2770 patients (3.7%) had an LRR and 7 033 (9.6%) a distant recurrence. The 5- and 10-year DRFS rates were 90.7% (95% c.i. 90.5 to 90.9) and 84.1% (83.7 to 84.5) for patients without a major systemic infection within 90 days of surgery, compared to 84.4% (82.1 to 86.4) and 76.3 (72.6 to 79.6) for patients with a major systemic infection.

**Fig. 2:**
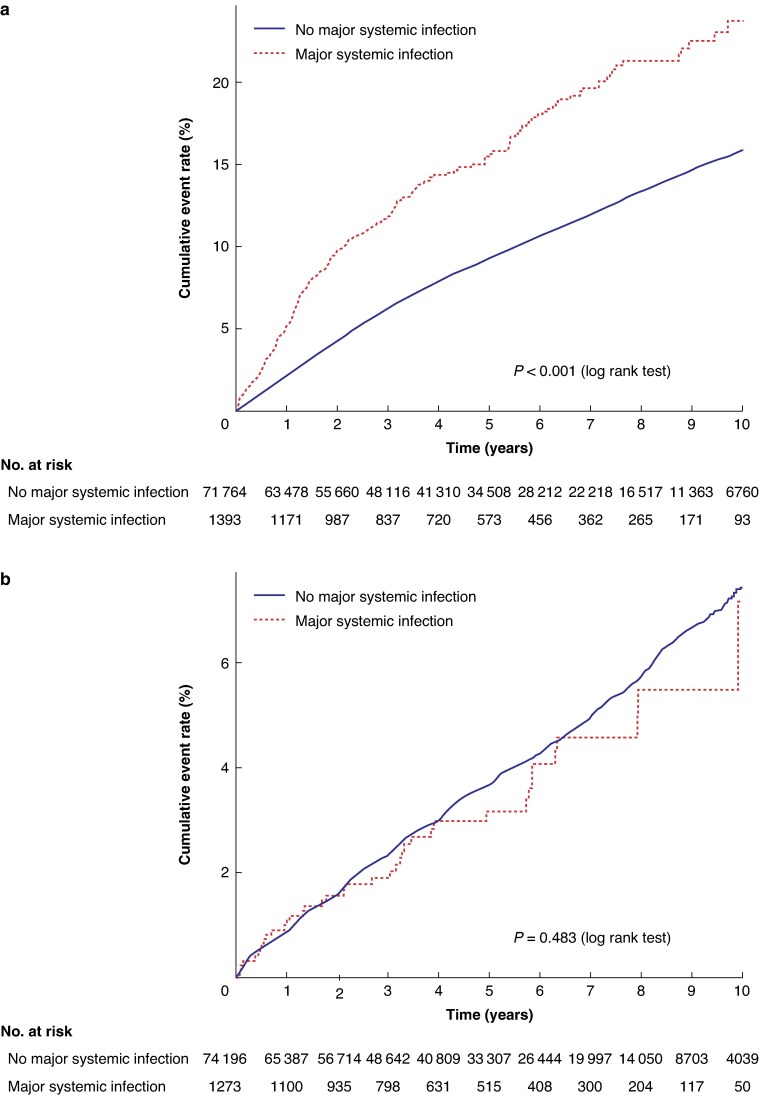

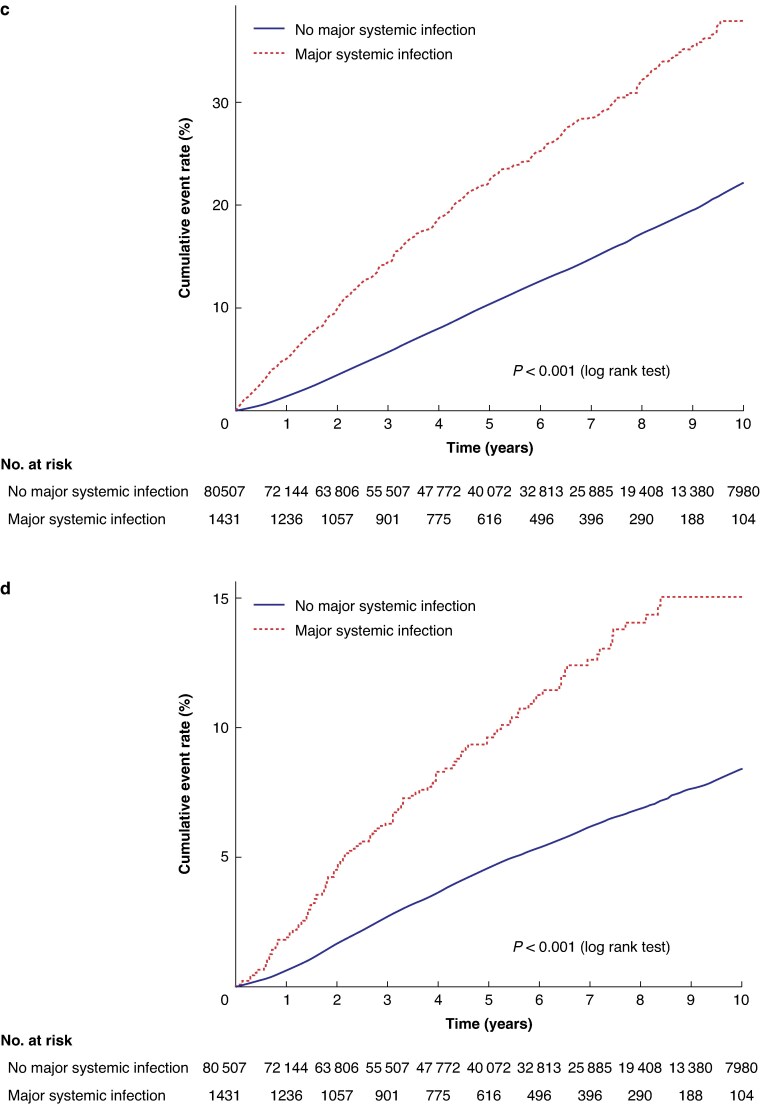
Kaplan–Meier analysis in patients with and without major systemic infection within 90 days

In the unadjusted analysis, the risks for all events except LRR were significantly increased after a major systemic infection within 90 days of surgery (distant recurrence: HR 1.78 (1.55–2.04), *P* < 0.001; LRR: HR 0.89 (0.65–1.22), *P* = 0.483; overall death: HR 2.20 (1.97–2.45), *P* < 0.001; and breast cancer-specific death: HR 2.17 (1.82–2.58), *P* < 0.001) (*Fig. 2a–d*). After adjustment for patient characteristics, disease characteristics, co-morbidity, socioeconomic factors, surgical and oncological treatment, the occurrence of a major systemic infection within 90 days was still significantly associated with higher rates of distant recurrence (HR 1.23 (1.07–1.41), *P* = 0.003), overall death (HR 1.47 (1.32–1.64), *P* < 0.001), and breast cancer-specific death (HR 1.27 (1.06–1.51), *P* = 0.008), but not with LRR (*Table 3*).

**a** Distant recurrence. **b** Locoregional recurrence. **c** Overall death. **d** Breast cancer-specific death.

**Table 3:** Stepwise adjusted Cox regression analysis of the association between major systemic infection (SI) and/or major event and time to distant recurrence, locoregional recurrence (LRR), overall death and breast cancer-specific death

Endpoint	Hazard ratio (95% c.i.)						
	Model 1	Model 2	Model 3	Model 4	Model 5	Model 6	Model 7
**Distant recurrence (*n* = 73 157)**							
Major SI							
early within 90 days Major event	2.37 (1.83–3.05)	1.96 (1.52–2.53)	1.72 (1.34–2.22)	1.68 (1.30–2.17)	1.67 (1.29–2.15)	1.69 (1.31–2.18)	1.66 (1.29–2.14)
	1.78 (1.55–2.04)	1.70 (1.48–1.95)	1.23 (1.07–1.41)	1.21 (1.06–1.39)	1.21 (1.05–1.38)	1.20 (1.05–1.38)	1.23 (1.07–1.41)
	1.86 (1.47–2.35)	1.64 (1.30–2.08)	1.27 (1.00–1.60)	1.24 (0.98–1.58)	1.23 (0.97–1.56)	1.25 (0.98–1.58)	1.24 (0.98–1.57)
**LRR (*n* = 75 469)**							
Major SI							
early within 90 days Major event	1.76 (1.08–2.88)	1.83 (1.12–2.99)	1.90 (1.16–3.11)	1.88 (1.15–3.08)	1.85 (1.13–3.03)	1.92 (1.17–3.14)	1.84 (1.12–3.02)
	0.89 (0.65–1.22)	0.91 (0.66–1.24)	0.96 (0.70–1.33)	0.96 (0.70–1.31)	0.95 (0.69–1.31)	0.97 (0.71–1.33)	0.99 (0.72–1.36)
	0.74 (0.40–1.38)	0.78 (0.42–1.46)	0.82 (0.44–1.52)	0.81 (0.44–1.51)	0.82 (0.44–1.52)	0.82 (0.44–1.53)	0.87 (0.46–1.61)
**Overall death (*n* = 81 938)**							
Major SI							
early within 90 days Major event	3.47 (2.88–4.16)	2.05 (1.71–2.47)	1.93 (1.61–2.33)	1.82 (1.52–2.19)	1.80 (1.50–2.17)	1.83 (1.52–2.21)	1.79 (1.49–2.15)
	2.20 (1.97–2.45)	1.87 (1.68–2.08)	1.55 (1.39–1.72)	1.48 (1.33–1.65)	1.47 (1.32–1.64)	1.45 (1.30–1.62)	1.47 (1.32–1.64)
	2.97 (2.52–3.51)	1.95 (1.65–2.30)	1.64 (1.39–1.94)	1.61 (1.37–1.91)	1.58 (1.34–1.86)	1.62 (1.37–1.91)	1.62 (1.37–1.91)
**Breast cancer-specific death (*n* = 81 938)**							
Major SI							
early within 90 days Major event	2.99 (2.18–4.10)	2.22 (1.62–3.05)	1.87 (1.36–2.57)	1.84 (1.34–2.52)	1.82 (1.32–2.49)	1.88 (1.37–2.59)	1.86 (1.35–2.55)
	2.17 (1.82–2.58)	1.98 (1.67–2.36)	1.25 (1.05–1.49)	1.25 (1.05–1.49)	1.24 (1.04–1.48)	1.25 (1.05–1.48)	1.27 (1.06–1.51)
	2.10 (1.54–2.86)	1.73 (1.27–2.36)	1.20 (0.88–1.64)	1.19 (0.87–1.62)	1.16 (0.85–1.58)	1.20 (0.87–1.64)	1.19 (0.87–1.62)

Model 1: Unadjusted; Model 2: Adjusted for patient characteristics (age, year of surgery, and region of residence); Model 3: +disease characteristics (T stage, subtype, N stage and histological tumour type); Model 4: +co-morbidity (CCI); Model 5: +socioeconomy (country of birth, highest level of education, and family income); Model 6: +surgical treatment (type of primary treatment, final breast/axillary surgery, and number of surgeries); Model 7: +oncological treatment (RT, chemotherapy, endocrine therapy and anti-HER2 therapy). SI: Systemic infection; CCI: Charlson Comorbidity Index; RT: radiotherapy.

The sensitivity analysis, excluding all patients with any type of malignancy before the breast cancer diagnosis (*n* = 7 415), did not alter these results (distant recurrence: HR 1.21 (1.04–1.40), *P* = 0.012; overall death: HR 1.45 (1.29–1.64), *P* < 0.001; and breast cancer-specific death: HR 1.28 (1.07–1.54), *P* = 0.008).

The adjusted outcomes for patients who experienced early (within 30 days) major systemic infection were even more pronounced (distant recurrence (HR 1.66 (1.29–2.14), *P* < 0.001), overall death (HR 1.79 (1.49–2.15), *P* < 0.001), and breast cancer-specific death (HR 1.86 (1.35–2.55), *P* < 0.001)). Additionally, these patients had a significantly increased risk of LRR (HR 1.84 (1.12–3.02), *P* = 0.015) (*Table 3*).

After adjustment, patients with other major events within 90 days did not have a significantly higher risk of distant recurrence (HR 1.24 (0.98–1.57), *P* = 0.074), LRR (HR 0.87 (0.46–1.61), *P* = 0.650), or breast cancer-specific death (HR 1.19 (0.87–1.62), *P* = 0.286). They did, however, have a higher risk of overall death (1.62 (1.37–1.91), *P* < 0.001) (*Table 3*).

## Discussion

In this large nationwide cohort study, a major systemic infection within 90 days of surgery requiring hospital admission was associated with a higher risk of distant recurrence, as well as overall and breast cancer-specific death. Patients with early major systemic infections had an even higher risk of all outcomes, including LRR. Conversely, patients with other major events only had an increased risk of overall death. The finding that major infection is associated with worse oncological outcomes aligns with a previously published Swedish study by de Boniface *et al*.^6^, which demonstrated that patients with major postoperative local complications within 30 days of surgery had worse OS and BCSS. However, in a related study involving the same cohort as the present analysis, SSIs were not associated with an increased risk of recurrence or death^18^. Interestingly, patients who experienced other unspecified local complications (such as implant-related issues, accidental puncture or injuries during surgery, retained foreign bodies, or other non-specific surgical complications) had a higher risk of distant recurrence (HR 1.22 (1.06–1.40)). Among these unspecified complications, 35.5% (590 of 1 663) were classified as major, requiring readmission or surgery due to the complication, whereas only 8.3% (1 072 of 12 875) of the SSIs were classified as major.

It is reasonable to assume that a major complication leads to a significantly greater inflammatory response, which may contribute to poorer outcomes. Additionally, the systemic inflammatory response to a major systemic infection could affect the residual microscopic disease, potentially promoting recurrence. Another factor to consider is that major complications are more likely to delay adjuvant oncological treatment. In the present study, patients with a major systemic infection had a significantly longer time to RT than those without such an infection. Although data on the time to chemotherapy were unavailable for analysis, it is reasonable to assume that chemotherapy may also be postponed in the event of a major infection. Moreover, it was impossible to account for all variables, leaving residual confounding. For example, a predisposition to infections could also indicate an underlying vulnerability that reduces the efficacy of, or response to, adjuvant therapy, ultimately leading to worse oncological outcomes.

The perioperative period has been identified as critical for determining oncological outcomes^21,29–31^. During this brief timeframe, factors such as heightened stress, inflammatory responses, and pro-angiogenic substances and/or growth factors may contribute to progression of pre-existing micrometastases^22,29^. Adjuvant therapy, which aims to eradicate residual microscopic disease and reduce the risk of recurrence and death^32^, is typically not initiated earlier than 1 month after surgery to allow wound healing. Data suggest that delays in adjuvant treatment increase the risk of recurrence and death^33–35^, and postoperative complications may further delay the initiation of RT^18^ as was observed in the current study. In 1863, Rudolf Virchow discovered white blood cells in malignant tissues and concluded that there was a link between cancer and inflammation^36^. This theory is now widely accepted^24,36^, with evidence suggesting that as much as 20% of all cancer-related deaths may be associated with infection and inflammation^37^.

Breast cancer recurrence can occur after prolonged latency periods, ranging from years to decades. One proposed explanation is cancer dormancy, a stage in which residual disease remains clinically undetectable and asymptomatic^38–40^. The perioperative period is a pivotal phase that can influence subsequent oncological outcomes^21^. Studies suggest that perioperative cyclooxygenase-2 and β-adrenergic blockade may be beneficial for breast cancer patients by reducing systemic inflammation and inhibiting metastasis-related pathways^22,30,31^. Other researchers have proposed that immunomodulatory interventions during the perioperative period could also improve survival outcomes^22,29^.

To the best of the authors knowledge, no previous study has investigated the association between postoperative major systemic infections and recurrence. Existing evidence regarding the potential association between SSI and distant recurrence remains inconclusive^1–6,12–17^. One possible explanation for the observed association in this study, between major systemic infection and recurrence and death, in contrast to the lack of such association with SSI^18^, is the significantly more extensive systemic inflammatory response triggered by conditions such as pneumonia than SSI. Moreover, defining SSI is challenging, and it is likely that some patients classified as having SSI may not actually have this condition. In contrast, diagnoses of systemic infections such as pneumonia are generally more reliable.

The main strengths of the current study include its large sample size, the national population-based setting with high-validity registries, substantially complete coverage, and complete follow-up. With the National Patient Register capturing about 99% of all hospitalizations^26^, the absolute majority of patients with major systemic infections during the study period should be included. Additionally, the availability of comprehensive data on numerous potential confounders, where the large size of the current study enabled robust adjustments for these factors in the multivariable analysis.

The study also has certain limitations. Data on systemic oncological treatment were missing for approximately 13.6% of patients. This is mainly due to delayed reporting of oncological treatment to the NKBC for patients diagnosed during the last year of the study period. Although coverage of oncological treatment in the register is generally high (>98% for postoperative treatment during the years included^41^), there can be a reporting lag of up to 24 months^42^. The missing data are not believed to be systematically related to patient or tumour characteristics and are therefore unlikely to introduce substantial bias. Another potential limitation is the underreporting of distant recurrence and LRR, which might lead to underestimation of the true recurrence rate. In the current study, 9.6% of patients developed a distant recurrence, a figure consistent with findings from a study that applied machine learning to optimize the identification of metastatic breast cancer using various demographic and population-based Swedish healthcare registers^43^. According to that study, 7.5% of breast cancer patients developed a distant recurrence during the years 2009 and 2016. Regarding LRR, patients undergoing breast conserving surgery in the MINDACT trial had an 8-year cumulative LRR incidence of 3.2% (2.7–3.7)^44^, compared with 3.7% in the current study with a median follow-up of 4.5 years. Considering these figures, the reporting of recurrences in the current study was deemed adequate. A potentially more important limitation, however, is the lack of data on patient frailty and the reasons for omission or delay of adjuvant therapy. Although adjustments were made for age and co-morbidity, frail patients are likely at higher risk for systemic infections and less likely to receive guideline-concordant treatment, which can worsen oncological outcomes even without the effect of a systemic infection. Information on smoking habits, alcohol consumption, BMI and time to adjuvant chemotherapy were also missing, all of which may influence both the risk of complications and oncological outcomes. Finally, given the risk of late recurrence in ER-positive breast cancer, the median follow-up of 4.8 years is relatively short, yet important associations were shown.

This study shows that a major systemic infection within 90 days of surgery is associated with an increased risk of distant recurrence, overall and breast cancer-specific death. This underscores the importance of implementing strategies to prevent postoperative infections and, in the event of an infection, provide prompt and adequate treatment to prevent its progression to a major infection.

## Supplementary Material

znaf233_Supplementary_Data

## Data Availability

The data are not publicly available due to restrictions by Swedish and European law to protect patient privacy. Data are available from the register holder of BCBaSe 3.0 for researchers with relevant ethical approvals and who meet the criteria for access to confidential data.
