# Supplementary material for: Major systemic infection following breast cancer surgery and oncological outcomes
Source: Br J Surg. 2025 Dec 2;112(12):znaf233. doi: 10.1093/bjs/znaf233 (PMC12672026; doi:10.1093/bjs/znaf233)
Supplement: znaf233_Supplementary_Data [file znaf233_supplementary_data.docx]

**Title**

**Major systemic infection following breast cancer surgery and oncologic outcomes**

Authors

Linda Adwall, PhD^1,2^, Irma Fredriksson, PhD^3,4^, Hella Hultin, PhD^3^, Peter Stålberg, Prof ^6^, Maria Mani, Assoc Prof^5^, Olov Norlén, Assoc Prof^6*^, Helena Sackey, PhD ^3,4*^

^1^Department of Surgery, Division of breast surgery, Södersjukhuset, Stockholm, Sweden

^2^Deparment of Surgical Sciences, Uppsala University, Uppsala, Sweden

^3^Department of Molecular Medicine and Surgery, Karolinska Institutet, Stockholm, Sweden

^4^Department of Breast-, Endocrine Tumors and Sarcoma, Karolinska Comprehensive Cancer Center, Karolinska University Hospital, Stockholm, Sweden

^5^Department of Surgical Sciences, Section of Plastic Surgery, Uppsala University, Uppsala University Hospital, Sweden

^6^Department of Surgical Sciences, Uppsala University, Uppsala University Hospital, Sweden

*both authors contributed equally

**Corresponding author.**

Dr Linda Adwall

Address: Department of Surgical Sciences, Uppsala University, SE-75185, Uppsala, Sweden

e-mail: [linda.adwall@uu.se](mailto:linda.adwall@uu.se)

**ORCID ID**;

0000-0002-0880-2468

**Supplementary Materials - Index**

| **Supplementary Methods** |  |
| --- | --- |
| BCBaSe 3.0 | *pag. 3* |
| International Classification of Diseases (ICD) codes for distant and locoregional recurrence | *pag. 5* |
| **Supplementary Figures and Tables** |  |
| Figure S1; Description of the BCBaSe 3.0 register | *pag. 6* |
| Table S1; Treatment characteristics | *pag. 7* |
|  |  |
|  |  |

**Supplementary Methods**

BCBaSe 3.0

The study is based on data from Breast Cancer Database Sweden 3.0 (BCBase 3.0), which is a population-based nation-wide database including individuals diagnosed with breast cancer in Sweden 2008-2019, created for the purpose of facilitating population-based epidemiological breast cancer research. BCBaSe 3.0 is based on individual level record linkages between information in the Swedish National Breast Cancer Quality Register (NKBC) and national demographic and population-based health care registers held by the Swedish National Board of Health and Welfare (the National Cancer Register, the National Cause of Death Register, the National Patient Register, the National Prescribed Drug Register), by Statistics Sweden (the Total Population Register, the Multi-generation Register, the Longitudinal integration database for health insurance and labour market studies (LISA)) and by the Swedish Social Insurance Agency (the Micro Data for Analysis of Social insurance (MIDAS)).

The NKBC contain detailed clinical data on patient and tumor characteristics, treatment and follow-up. The completeness of the NKBC is high, >99%, assessed by cross-linkage to the National Cancer Register to which reporting is mandatory by law. The proportion of missing values is <5% for most variables and reported information generally has high exact concordance (1). The National Cancer Registry records data on all cancer diagnoses including site and date, ICD code, morphological SNOMED code and base for diagnosis. The register is estimated to cover more than 96–98% of all incident malignant tumors in Sweden, and with 98% of the diagnoses being morphologically verified (2, 3). The National Cause of Death register records information on date of death, underlying and contributing cause(s) of death according to ICD (4). Overall, 96% of individuals in the Cause of Death Register have a specific cause of death recorded. For breast cancer the accuracy of death certificates is estimated to be 93.1% (5). The National Patient Register includes information on in- and outpatient patient care with up to eight discharge diagnosis classified according to ICD, data on surgical procedures, dates of admission and discharge. The register is estimated to capture about 99% of all hospitalizations (6). The National Prescribed Drug Register comprises information on all prescribed drugs dispensed in Swedish pharmacies classified according to the Anatomic Therapeutical Chemical (ATC) classification system including dates of dispensation and number of defined daily doses (DDD) (7, 8). The Total Population Register includes information on vital status (alive/dead), place of residence, country of birth, immigration and emigration (9). The LISA database contains individual level information on socioeconomic variables such as marital status, highest achieved educational level, disposable income, profession, housing type, country of birth and parents’ country of birth (10, 11). Data from the Multi-generation Register and the MIDAS database were not used for the present study.

1. Löfgren L, Eloranta S, Krawiec K, Asterkvist A, Lönnqvist C, Sandelin K. Validation of data quality in the Swedish National Register for Breast Cancer. BMC Public Health. 2019;19(1):495.

2. Mattsson B, Wallgren A. Completeness of the Swedish Cancer Register. Non-notified cancer cases recorded on death certificates in 1978. Acta Radiol Oncol. 1984;23(5):305-13.

3. Barlow L, Westergren K, Holmberg L, Talbäck M. The completeness of the Swedish Cancer Register: a sample survey for year 1998. Acta Oncol. 2009;48(1):27-33.

4. Brooke HL, Talbäck M, Hörnblad J, Johansson LA, Ludvigsson JF, Druid H, et al. The Swedish cause of death register. Eur J Epidemiol. 2017;32(9):765-73.

5. Nyström L, Larsson LG, Rutqvist LE, Lindgren A, Lindqvist M, Rydén S, et al. Determination of cause of death among breast cancer cases in the Swedish randomized mammography screening trials. A comparison between official statistics and validation by an endpoint committee. Acta Oncol. 1995;34(2):145-52.

6. Ludvigsson JF, Andersson E, Ekbom A, Feychting M, Kim JL, Reuterwall C, et al. External review and validation of the Swedish national inpatient register. BMC Public Health. 2011;11:450.

7. Socialstyrelsen. The Swedish Prescribed Drug Register: Socialstyrelsen; 2020 [08 Jan 2021]. Available from: <https://www.socialstyrelsen.se/en/statistics-and-data/registers/national-prescribed-drug-register/>

8. Wettermark B, Hammar N, Fored CM, Leimanis A, Otterblad Olausson P, Bergman U, et al. The new Swedish Prescribed Drug Register--opportunities for pharmacoepidemiological research and experience from the first six months. Pharmacoepidemiol Drug Saf. 2007;16(7):726-35.

9. <https://www.scb.se/contentassets/8f66bcf5abc34d0b98afa4fcbfc0e060/rtb-bar-2016-eng.pdf>.

10. Statistics Sweden. Longitudinal integrated database for health insurance and labour market studies: Statistics Sweden; [08 Jan 2021]. Available from: <https://www.scb.se/en/services/ordering-data-and-statistics/register/longitudinal-integrated-database-for-health-insurance-and-labour-market-studies-lisa/>

11. Ludvigsson JF, Svedberg P, Olén O, Bruze G, Neovius M. The longitudinal integrated database for health insurance and labour market studies (LISA) and its use in medical research. Eur J Epidemiol. 2019;34(4):423-37.

International Classification of Diseases (ICD) codes for distant and locoregional recurrence

**International Classification of Diseases (ICD) codes for distant recurrence**

C78.0 Secondary malignant tumor (metastasis) in the lung

C78.1 Secondary malignant tumor (metastasis) in the mediastinum

C78.2 Secondary malignant tumor (metastasis) in the pleura

C78.3 Secondary malignant tumor (metastasis) in other and unspecified organs

C78.4 Secondary malignant tumor (metastasis) in the small intestine, including the duodenum

C78.5 Secondary malignant tumor (metastasis) in the colon and rectum

C78.6 Secondary malignant tumor (metastasis) in the retroperitoneal space and peritoneum

C78.7 Secondary malignant tumor (metastasis) in the liver and intrahepatic bile ducts

C78.8 Secondary malignant tumor (metastasis) in other and unspecified digestive organs

C79.0 Secondary malignant tumor (metastasis) in the kidney and renal pelvis

C79.1 Secondary malignant tumor (metastasis) in the bladder and other unspecified urinary organs

C79.3 Secondary malignant tumor (metastasis) in the brain and meninges

C79.4 Secondary malignant tumor (metastasis) in other and ospecified parts of the nervous system

C79.5 Secondary malignant tumor (metastasis) in the bones and bone marrow

C79.6 Secondary malignant tumor (metastasis) in the ovary

C79.7 Secondary malignant tumor (metastasis) in the adrenal gland

C79.8 Secondary malignant tumor (metastasis) in other unspecified locations

C79.9 Secondary malignant tumor (metastasis) in unspecified locations

C77.1 Secondary malignant tumor (metastasis) in intrathoracic lymph nodes

C77.2 Secondary malignant tumor (metastasis) in intra-abdominal lymph nodes

C77.8 Secondary malignant tumor (metastasis) in lymph nodes in multiple body regions

**ICD codes for locoregional recurrence**

C50 Malignant tumor of the breast

D05 Cancer in situ of the breast (excluding D05.0 lobular carcinoma in situ (LCIS))

C79.2 Secondary malignant tumor in the skin

C77.0 Secondary malignant tumor (metastasis) in lymph nodes of the head, face, or neck

C77.3 Secondary malignant tumor (metastasis) in lymph nodes in the axilla and upper extremity

C77.9 Secondary malignant tumor (metastasis) in lymph nodes

**Supplementary Figures and Tables**

Figure S1; Description of the BCBaSe 3.0 register

**
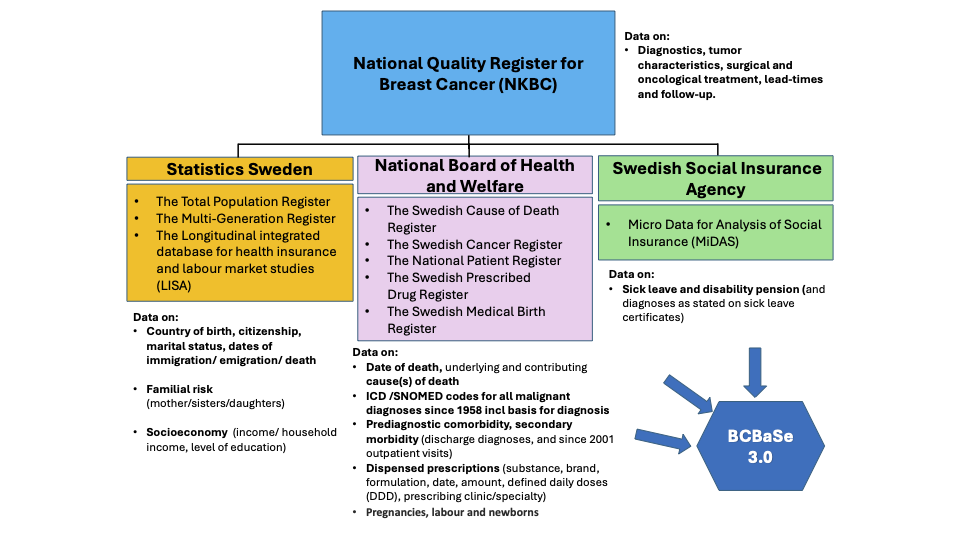
**

**Table S1. Treatment characteristics, major systemic infection within 90 days,**

**in a population-based cohort of 82 102 individuals with breast cancer**

**diagnosed between 2008 and 2019.**

|  | **No infection**  **n=80 641 (%)** | **Infection**  **n=1 461 (%)** | **Overall**  **n=82 102 (%)** |
| --- | --- | --- | --- |
| **Mode of detection**  Screening  Clinical  Missing | 42855 (53.2)  37519 (46.6)  227 (0.3) | 574 (39.3)  884 (60.6)  2 (0.1) | 43429 (52.9)  38403 (46.8)  229 (0.3) |
| **Laterality**  Right  Left | 39167 (48.6)  41474 (51.4) | 713 (48.8)  748 (51.2) | 39880 (48.6)  42222 (51.4) |
| **Year of surgery**  2008-2010  2011-2013  2014-2016  2017-2019 | 18971 (23.5)  20855 (25.9)  21219 (26.3)  19596 (24.3) | 342 (23.4)  386 (26.4)  390 (26.7)  343 (23.5) | 19313 (23.5)  21241 (25.9)  21609 (26.3)  19939 (24.3) |
| **Region of residence**  Stockholm/Gotland  Uppsala/Orebro  North  South  Southeast  West  Missing | 17779 (22.1)  17184 (21.3)  7054 (8.8)  14001 (17.4)  8331(10.3)  16041 (19.9)  251 (0.3) | 327 (22.4)  328 (22.5)  172 (11.8)  248 (17.0)  143 (9.8)  243 (16.6)  0 (0.0) | 18106 (22.1)  17512 (21.3)  7226 (8.8)  14249 (17.4)  8474 (10.3)  16284 (19.8)  251 (0.3) |
| **Primary treatment**  Surgery  NAT  Missing | 75393 (93.5)  5164 (6.4)  84 (0.1) | 1351 (92.5)  106 (7.3)  4 (0.3) | 76744 (93.5)  5270 (6.4)  88 (0.1) |
| **Final breast surgery**  BCS  Mastectomy  Mastectomy + IBR  Only axillary surgery  Missing | 48943 (60.7)  28420 (35.2)  3038 (3.8)  183 (0.2)  57 (0.1) | 667 (45.7)  735 (50.3)  45 (3.1)  11 (0.8)  3 (0.2) | 49610 (60.4)  29155 (35.5)  3083 (3.8)  194 (0.2)  60 (0.1) |
| **Final axillary surgery**  SLNB  ALND  Sampling  Missing | 52383 (65.0)  21627 (26.8)  1230 (1.5)  5401 (6.7) | 744 (50.9)  631 (43.2)  23 (1.6)  63 (4.3) | 53127 (64.7)  22258 (27.1)  1253 (1.5)  5464 (6.7) |
| **Number of surgeries^#^**  1  2  3  4  Missing | 67652 (83.9)  10340 (12.8)  653 (0.8)  38 (0.1)  1958 (2.4) | 1290 (88.3)  127 (8.7)  9 (0.6)  1 (0.1)  34 (2.3) | 68942 (84.0)  10467 (12.8)  662 (0.8)  39 (0.1)  1992 (2.4) |
| **Radiotherapy**  No  Yes  Missing | 19536 (24.2)  49693 (61.6)  11412 (14.2) | 392 (26.8)  859 (58.8)  210 (14.4) | 19928 (24.3)  50552 (61.6)  11622 (14.2) |
| **Time to radiotherapy**  $\leq$60 days  61-90 days  >90 days  Missing | 13689 (17.0)  12773 (15.8)  23104 (28.7)  31075 (38.5) | 100 (6.8)  94 (6.4)  662 (45.3)  605 (41.4) | 13789 (16.8)  12867 (15.7)  23766 (29.0)  31680 (38.6) |
| **Chemotherapy^##^** |  |  |  |
| No  Yes  Missing | 49888 (61.9)  19779 (24.5)  10974 (13.6) | 550 (37.7)  710 (48.6)  201 (13.8) | 50438 (61.4)  20489 (25.0)  11175 (13.6) |
| **Endocrine treatment**  No  Yes  Missing | 21248 (26.4)  48048 (59.6)  11345 (14.1) | 360 (24.6)  893 (61.1)  208 (14.2) | 21608 (26.3)  48941 (59.6)  11553 (14.1) |
| **Anti-HER2 therapy**  No  Yes  Missing | 62385 (77.4)  6812 (8.5)  11444 (14.2) | 1018 (69.7)  231 (15.8)  212 (14.5) | 63403 (77.2)  7043 (8.6)  11656 (14.2) |

Values are number (percent). Percentages may not add up to 100 percent owing rounding.

^#^breast/axilla due to tumour data ^##^adjuvant. NAT neoadjuvant therapy; BCS breast conserving surgery;

IBR Immediate breast reconstruction; SLNB sentinel lymph node biopsy; ALND axillary lymph node dissection.
